# Defense Responses in Rice Induced by Silicon Amendment against Infestation by the Leaf Folder *Cnaphalocrocis medinalis*

**DOI:** 10.1371/journal.pone.0153918

**Published:** 2016-04-28

**Authors:** Yongqiang Han, Pei Li, Shaolong Gong, Lang Yang, Lizhang Wen, Maolin Hou

**Affiliations:** 1 State Key Laboratory for Biology of Plant Diseases and Insect Pests, Institute of Plant Protection, Chinese Academy of Agricultural Sciences, Beijing, 100193, China; 2 Southern Regional Collaborative Innovation Center for Grain and Oil Crops in China, Changsha, 410128, China; 3 College of Plant Protection, Hunan Agricultural University, Changsha, 410128, China; Universidade Federal de Viçosa, BRAZIL

## Abstract

Silicon (Si) amendment to plants can confer enhanced resistance to herbivores. In the present study, the physiological and cytological mechanisms underlying the enhanced resistance of plants with Si addition were investigated for one of the most destructive rice pests in Asian countries, the rice leaf folder, *Cnaphalocrocis medinalis* (Guenée). Activities of defense-related enzymes, superoxide dismutase, peroxidase, catalase, phenylalanine ammonia-lyase, and polyphenol oxidase, and concentrations of malondialdehyde and soluble protein in leaves were measured in rice plants with or without leaf folder infestation and with or without Si amendment at 0.32 g Si/kg soil. Silicon amendment significantly reduced leaf folder larval survival. Silicon addition alone did not change activities of defense-related enzymes and malondialdehyde concentration in rice leaves. With leaf folder infestation, activities of the defense-related enzymes increased and malondialdehyde concentration decreased in plants amended with Si. Soluble protein content increased with Si addition when the plants were not infested, but was reduced more in the infested plants with Si amendment than in those without Si addition. Regardless of leaf folder infestation, Si amendment significantly increased leaf Si content through increases in the number and width of silica cells. Our results show that Si addition enhances rice resistance to the leaf folder through priming the feeding stress defense system, reduction in soluble protein content and cell silicification of rice leaves.

## Introduction

The rice leaf folder (LF), *Cnaphalocrocis medinalis* Guenée (Lepidoptera: Pyralidae), one of the most devastating migratory insect pests of rice, is widely distributed in humid tropical and temperate regions of Asia, Oceania, Australia and Africa, between 48°N and 24°S and 0°E to 172°W [1]. The insect migrates to China from the Sino-India peninsula in the spring annually. The leaf folder larvae damage the rice plant by folding leaves and scraping green leaf tissues within the fold, causing severe yield losses by reducing photosynthetic activity [2]. Recently, it has become widespread throughout the major rice-growing regions of Asia [3]. The annual average area infested by LF in China has been more than 20 million ha, causing yield loss of more than 700 million kg each year [3].

To reduce the yield loss due to LF infestation, conventional chemical control has been employed, which is expensive and laborious, and leads to environmental pollution. In addition, overuse of pesticides destroys natural enemies and leads to the insect developing resistance, which results in pest resurgence [4]. Hence, there is a need to search for alternative ways for the control of this pest. Cultivar resistance and crop management are key tactics currently being developed.

Silicon (Si) addition is one of the alternative methods to chemical control of insect pests. Although Si is generally not considered an essential element for plant growth, due to its important role when the plants are subjected to abiotic and/or biotic stresses, it is now recognized as a “beneficial substance” or “quasi-essential” [5,6]. There is increasing evidence showing positive relationships between high plant Si content and plant resistance to insect herbivory in both monocot and dicot plants [7]. Enhanced herbivory resistance resulting from Si amendment has been reported in several susceptible Poaceous crop varieties [8–12] and grasses [13,14].

Silicon may mediate plant defense against insect herbivores in several ways: (1) indirect defense based on augmented release of herbivore-induced plant volatiles (HIPVs) that attract natural enemies of the attacking herbivores [15]; (2) direct defense through increased physical (passive) resistance due to amorphous silica deposited in plant tissues, leading to reduced digestibility and/or increased hardness and abrasiveness in plants [13,14]; (3) differential regulation of genes, as indicated in powdery mildew-stressed *Arabidopsis* plants [16] and in rice plants infected by the rice blast fungus [17], which may also occur in responses of Si-amended plants to insect herbivory; (4) active priming of plant chemical defenses by soluble Si and its interaction with the jasmonate signaling pathway, facilitating production of defensive enzymes such as catalase (CAT), peroxidase (POD), superoxide dismutase (SOD), polyphenol oxidase (PPO) and phenylalanine ammonia-lyase (PAL), which are key enzymes regulating the production and accumulation of secondary metabolic compounds, such as phenolics, phytoalexins, and momilactones [18,19]. This Si-mediated resistance mechanism has been well documented in plant defense to pathogens.

Reactive oxygen species (ROS) are common components of the defense responses of plants against pathogen and herbivore attacks [20], while excessive levels of ROS can cause significant damage to cell structures [21]. Plants protect themselves from cytotoxic effects of these ROS with SOD, POD and CAT [20]. SOD removes superoxide anion free radicals accompanying the formation of H_2_O_2_, which is then detoxified by POD and CAT [22]. PAL is involved in the biosynthesis of phenolics, phytoalexins and lignins [23]. PPO and POD are oxidases that catalyze the formation of lignin and other oxidative phenols [24]. PPO catalyzes oxidation of phenols to quinines that can restrict development of herbivorous insects [25]. Polyunsaturated fatty acids (PUFAs) peroxides generate malondialdehyde (MDA), the most abundant aldehydic lipid breakdown product that indicates the levels of stress and injury to the functional membrane [26].

Particularly, studies have revealed that Si plays an active role in priming plant defense against herbivores. In Si-amended rice plants, we found enhanced resistance to LF due to reduced food conversion efficiency [11] and impaired feeding behavior [27]. Ye et al. [19] showed that LF infestation enhanced activities of enzymes (POD and PPO) for secondary metabolic compounds in rice plants amended with Si. However, the possibility of Si-induced activity changes in defense-related enzymes, including both antioxidative enzymes and enzymes for secondary metabolic compounds, in plants infested by LF has yet to be investigated. Plant soluble protein is the main N source for herbivores, but the effects of Si amendment on plant protein concentration are not clear. Answers to these latter problems can help in understanding the physiological bases for Si-mediated rice plant resistance to LF.

Our overall hypothesis was that Si amendment would amplify the defense responses in rice plant to LF attack. Specifically, we wanted to: (i) determine the interactive effects between Si addition and LF infestation on plant resistance to LF, (ii) identify whether Si addition induces activity changes in defense-related enzymes (SOD, POD, CAT, PAL and PPO) and MDA and soluble protein contents in response to LF infestation, and (iii) measure if Si addition intensifies cell silicification in rice. Such information may provide greater insight into Si-mediated resistance to LF and improve management of the insect pest in rice.

## Materials and Methods

### Plants, Si Treatments and Insects

The plants and Si treatments were largely the same as those reported by Han et al. [11]. Briefly, germinated seeds of a susceptible variety (Taichung Native 1, TN1) were sown in soil without addition of calcium silicate. Rice seedlings were transplanted to 10 L PVC pots 25 d after sowing at two 2-seedling hills per pot in a glasshouse at Guilin Experiment Station for Crop Pests (25°36'00" N, 110°41'24" E), Ministry of Agriculture, China.

The pots each were filled with 4.2 kg dry soil, amended with calcium silicate (soluble Si ≥ 11.7%, Shanxi Fubon Siliconfat Co., Ltd, Jinzhong, China) at a rate of 0.32 g Si/kg soil (+Si) or left un-amended (-Si). Soil used for the plants was obtained from the fields of the station, whose chemical properties were described previously [11]. All the pots were treated with urea (N ≥ 46.4%), diammonium phosphate (N = 16.0%; P_2_O_5_ = 44.0%) and potassium chloride (K_2_O ≥ 60.0%) at a rate of 0.37 g/kg soil, 0.25 g/kg soil and 0.35 g/kg soil, respectively. Application regime of the fertilizers and calcium silicate was the same as previously described [9,11]. The pots were arranged randomly in the glasshouse. Watering was administered as necessary and water level in the pots was always below the upper edge. Pesticides were not used throughout the experiment. Rice plants at 40 days after transplanting (DAT) were used in the experiments.

Rice leaf folder adults were collected in late June, 2014 from paddy fields at the experiment station. The adults were confined to caged rice plants in the field for oviposition. Eggs together with segments of leaf blades were collected daily from the plants and placed on moistened filter paper in a Petri dish (15 cm in diameter and 2 cm in height). Newly hatched first instars were used in the experiments or maintained on rice plants without Si addition in a climate chamber (RXZ-160A, Ningbo Dongnan Instruments Co., Ltd, Ningbo, China) at 28 ± 1°C, 70 ± 5% relative humidity (RH) and a photoperiod of 16:8 (L:D) h.

### Larval Survival Rate

A leaf segment method [11] was employed to determine the effects of Si amendment on survival of LF larvae using reciprocal fourth leaf segments cut from 40-DAT plants. Twenty newly moulted LF larvae were transferred at five larvae per dish onto the leaf segments with a pointed fine camel hair brush. The insects were left to develop in the climate chamber. The dishes were observed after 72 h for surviving larvae. Individuals which did not respond to the touch of a camel hair brush or were in a moribund condition were considered dead. The observation was repeated 3 times for 20-insect groups. The tests were performed for each of the 1^st^, 2^nd^, 3^rd^, 4^th^ and 5^th^ instars.

### Plant Defensive Enzyme Activities and Concentrations of Malondialdehyde (MDA) and Soluble Protein

To analyze plant defensive enzyme activities and concentrations of malondialdehyde (MDA) and soluble protein in response to LF infestation and Si amendment, the +Si and -Si 40-DAT potted rice plants were each divided into two groups: one was exposed to LF third instars and another, not exposed. Thus the plants were in four treatments: (1) without Si addition and LF infestation (-Si-LF); (2) without Si addition and with LF infestation (-Si+LF); (3) with Si addition and without LF infestation (+Si-LF); (4) with Si addition and LF infestation (+Si+LF). For the LF exposure treatments, one third-instar larva starved for 2 h was transferred with a pointed fine camel hair brush to the reciprocal fourth leaf of a randomly selected tiller. Ten tillers per pot were infested with LF larvae. The potted plants, whether exposed to LF infestation or not, were each caged with 60 mesh gauze. At 24, 48, 72, and 96 h after LF infestation, the reciprocal fourth leaves were harvested from LF-infested or un-infested plants and immediately maintained in liquid nitrogen for later analysis. The following measurements were each repeated three times using different leaf samples.

To determine the CAT, POD and SOD activities, frozen leaf samples of a treatment (0.5 g) were homogenized with a mortar and pestle in an ice bath with 100 mM phosphate buffer saline (PBS, pH 7.4), containing 1 mM EDTA and 1% (w/v) polyvinylpyrolidone (PVP), at a 1:10 ratio (fresh weight of leaf sample/buffer volume). The crude homogenates were centrifuged at 12,000 rpm for 15 min at 4°C (Centrifuge 5417R, Eppendorf, Hamburg, Germany). The supernatant was used to determine the enzyme activities using diagnostic kits (Nanjing Jiancheng Biotechnology Institute, China). CAT activity was measured according to the ammonium molybdate spectrophotometric method, based on the fact that ammonium molybdate could rapidly terminate the H_2_O_2_ degradation reaction catalysed by CAT and react with the residual H_2_O_2_ to generate a yellow complex, which could be monitored by the absorbance at 405 nm [28]. One unit of CAT activity was defined as the amount that reduces the level of H_2_O_2_ by 1 μmol per second per mg protein. POD activity was measured by a spectrophotometer (UNICO, UV-2000, Shanghai, China) following the change of absorption at 420 nm due to guaiacol oxidation [29]. One unit of POD activity was defined as the amount that catalyzes 1 μg substrate per minute per mg protein. For determination of SOD activity, 2-(4-iodophenyl)-3-(4-nitrophenyl)-5-(2, 4-disulfophenyl)-2H-tetrazolium (WST -1) method was used [30]. WST-1 can couple with xanthine oxidase (XO) to generate superoxide anions (O_2_^–^) and formazan dye, which can be inhibited by SOD by catalysing O_2_^–^ into H_2_O_2_ and O_2_ [31]. Therefore, the SOD activity can be calculated by measuring the absorbance of formazan dye at 450 nm. One unit of SOD activity was defined as the amount that causes a 50% reduction in the absorbance at 450 nm by using a Multiskan Spectrum (Thermo Fisher Scientific Ltd., Finland).

Phenylalanine ammonia-lyase (PAL) activity was assayed according to the methods of Cai et al. [32], with minor modification. Leaf samples of a treatment (0.5 g) were homogenized with a mortar and pestle in an ice bath in 5 ml of 50 mM borate buffer (pH 8.8) containing 5.0 mM thioalcohol and 1 mM EDTA. The homogenate was centrifuged at 13,000 rpm for 10 min at 4°C. A reaction mixture containing 2 ml 50 mM borate buffer (pH 8.8) and 1.0 ml 20 mM L-phenylalanine was added to 0.2 mL of the crude homogenate. After incubation for 30 min at 40°C, the reaction was stopped by adding 0.25 ml of 5 M HCl. The increase in the absorbance at 290 nm due to the formation of *trans*-cinnamate was measured using the spectrophotometer. One unit of PAL activity was defined as the absorbance increase by 0.01 unit h^−1^.

Polyphenol oxidase (PPO) was extracted following the method of Cai et al. [32]. Leaf samples of a treatment (0.25 g) were homogenized in an ice bath in 5 ml of 50 mM borate buffer (pH 8.7) containing 5.0 mM sodium hydrogen sulfite and 0.1 g PVP. The homogenate was centrifuged at 13,000 rpm for 10 min at 4°C. PPO activity was determined by adding 0.1 ml of the centrifuge supernatant to 3 ml of a solution containing 50 mM potassium phosphate buffer (pH 6.5) and 0.5 ml of 0.15 mM catechol. The increase of absorbance was measured at 420 nm by using the spectrophotometer for 10 min at 30°C [33]. One PPO unit was expressed as the variation of absorbance at 420 nm of soluble protein per minute.

The MDA content was determined by the thiobarbituric acid method using a commercial MDA assay kit (Nanjing Jiancheng Bioengineering Institute, Nanjing, China) following the manufacturer’s protocols. This method is based on the reaction of MDA with thiobarbituric acid, forming stable thiobarbituric acid-reactive substances, which shows peak absorbance at 532 nm [34]. MDA concentration was recorded using the Multiskan Spectrum. The result was expressed as nmol/mg protein.

Concentration of soluble protein was determined by the Coomassie Blue method [35] using bovine serum albumin as a standard, and the absorbance of the samples was measured at 595 nm by using the Multiskan Spectrum.

### Determination of Si Content and Microscopy Observation of Silica Cells

The reciprocal fourth leaves harvested at 96 h after LF infestation or from unexposed plants were used in determination of Si content and observation for silica cells. For determination of Si content, the fresh leaves were killed at 105°C for 30 min and dried at 75°C to constant weight and then crushed with a food pulverizer and sieved with a 0.25-mm screen. Silicon contents were measured from the resulting leaf powder using the procedures of Dai et al. [36].

Morphology of silica cells on the leaf surface was observed using scanning electron microscopy (SEM) (Hitachi S-570, Japan). A fresh specimen (0.3–0.5 cm in length) of the undamaged part of the reciprocal fourth leaf was sampled and wiped with tissue paper to remove moisture. The leaf segment was fixed with 2.5% (v/v) glutaraldehyde in 0.1 M phosphate buffer solution (pH 7.4) for 2–3 h at 4°C, and then post-fixed with 1% (w/v) osmium tetroxide in the phosphate buffer solution for 30 min. Thereafter, the leaf segment was dehydrated through a graded ethanol series [50, 70, 80, 90 and 100% (v/v)], dried by a critical-point drying method with liquid CO_2_ and coated with metal and then loaded onto the SEM. Ten SEM pictures (at 100 × magnification) were randomly selected from each treatment for silica cell measurement. Rows of silica cells per 1 mm^2^ leaf area and numbers of silica cells per 1-mm row were counted. The length and width of silica cells were measured by Image J software (version 1.37, NIH, Bethesda, USA). Additionally, SEM pictures (at 300 × magnification) were obtained to illustrate the difference of silica cells of rice leaves in the treatments.

### Data Analysis and Statistics

All data in figures and tables are shown as means ± SE. For larval survival rate, we performed independent-samples t-test following arcsine square root transformation of the data to analyze the significant difference between +Si and -Si treatment. The data of enzyme activities and concentrations of MDA and soluble protein were subjected to three-way analysis of variance (ANOVA) for the effects of Si amendment, LF infestation, LF infestation time and the interactions between the three treatments. Leaf Si content and silica cell measurements were analyzed by two-way ANOVA for the effects of Si addition, LF infestation and their interactions. The means were separated by Tukey’s multiple range test (*P* = 0.05) or by independent-samples t-test for significant differences between treatments. All statistical analysis was performed using SPSS 16.0 (SPSS Inc, USA).

## Results

### Larval Survival Rate

**Fig 1:**
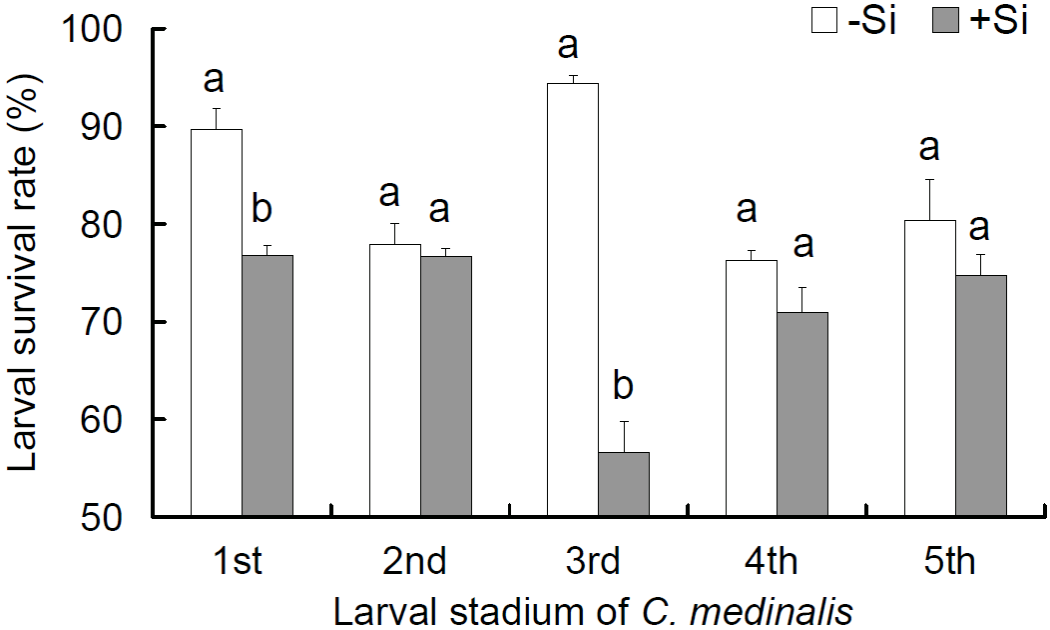
Effects of silicon amendment to rice plants on larval survival rate of *Cnaphalocrocis medinalis*. +Si = silicon amendment at 0.32 g Si/kg soil to rice plants,–Si = no silicon amendment. Values are means ± SE from 3 replicates measured with different leaf samples. Different letters over the bars in a certain larval stadium denote significant difference at *P* < 0.05 according to Independent-samples t-test.

Silicon amendment to rice plants significantly reduced survival rates of the first (Fig 1; *t* = 4.96, df = 4, *P* = 0.008) and third (*t* = 13.00, df = 4, *P* < 0.001) instars by 12.8% and 37.8%, respectively, as compared with the plants without Si addtion.

### Activities of Defense-Related Enzymes

#### SOD activity

**Fig 2:**
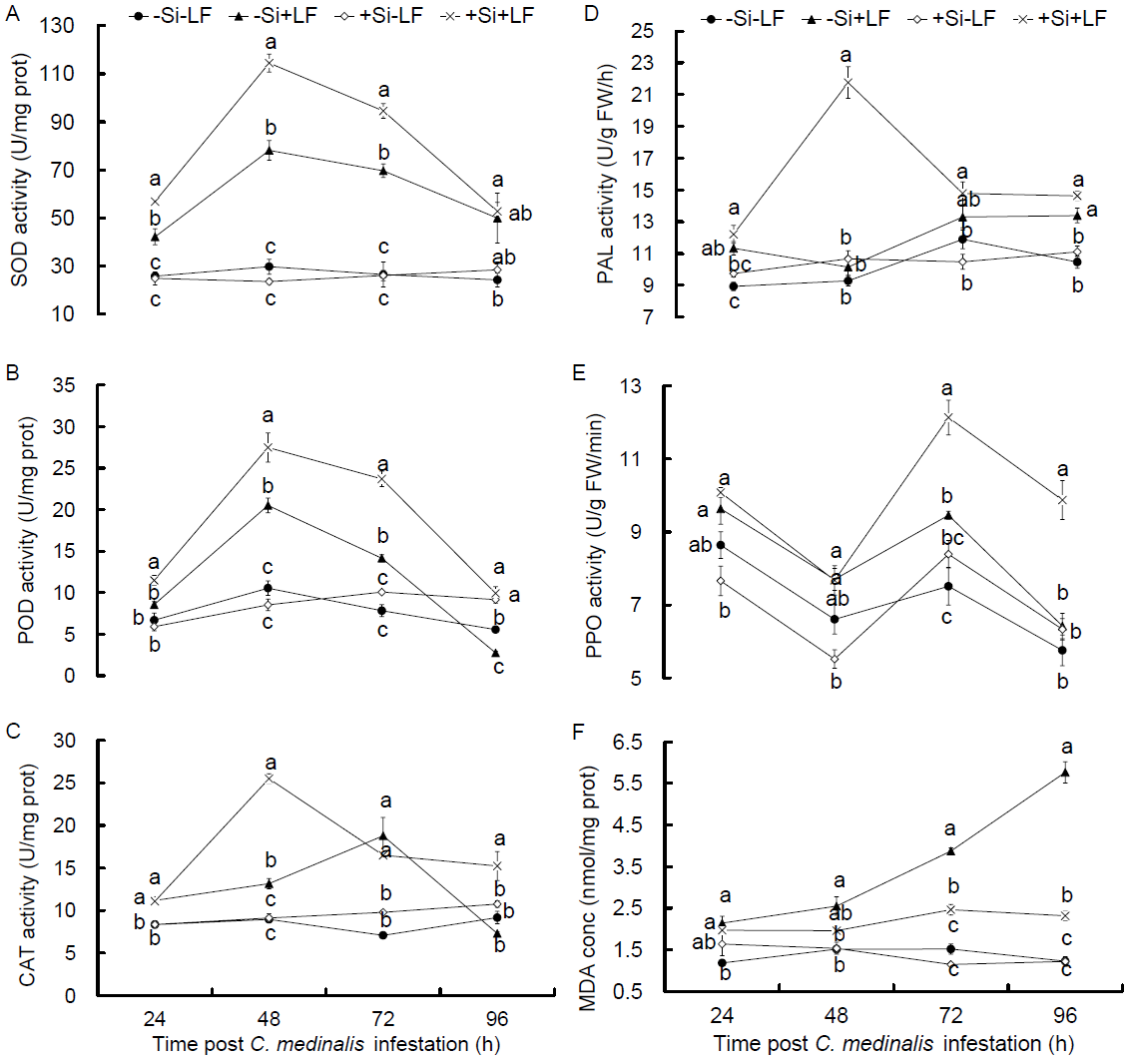
Effects of silicon amendment and *Cnaphalocrocis medinalis* infestation on activities of SOD (A), POD (B), CAT (C), PAL (D) and PPO (E) and concentration of MDA (F) in rice leaves. +Si = silicon amendment at 0.32 g Si/kg soil to rice plants,–Si = no silicon amendment. +LF = infestation of *C*. *medinalis* larvae,–LF = no infestation. Values are means ± SE from 3 replicates measured with different leaf samples. In each panel, the means labeled by different letters at a certain time point post *C*. *medinalis* infestation are significantly different at *P* < 0.05 according to Tukey’s multiple range tests.

ANOVA showed that Si addition, LF infestation and LF infestation time all significantly influenced SOD activity in leaves, the interactions between these factors also had significant effects with exception of the interaction between Si addition and LF infestation time (Table 1). Without LF infestation, SOD activity was not different between +Si and -Si plants at all time points (24–96 h post infestation) (Fig 2A; *t* ≤ 1.87, *P* ≥ 0.135). In infested plants, SOD activity increased over that in un-infested plants and showed a convex-shaped temporal pattern, the increases were significant except at 96 h post infestation (*F* ≥ 6.061, df = 3, 11, *P* ≤ 0.019). Silicon amendment added to the increase in SOD activity in infested plants, which is evidenced by higher increases (*t* ≥ 4.168, *P* ≤ 0.014) in +Si plants than in -Si plants by 34.7%, 46.5% and 35.8% at 24, 48 and 72 h post infestation, respectively.

**Table 1:** Three-way analysis of variance for significance (*P* value) of the effects of silicon amendment to plants, *Cnaphalocrocis medinalis* infestation and infestation time on enzyme activities and concentrations of malondialdehyde and soluble protein.

	SOD	POD	CAT	PAL	PPO	MDA	Soluble protein
Silicon amendment (A)	<0.001	<0.001	<0.001	<0.001	<0.001	<0.001	0.004
Infestation (B)	<0.001	<0.001	<0.001	<0.001	<0.001	<0.001	<0.001
Infestation time (C)	<0.001	<0.001	<0.001	<0.001	<0.001	<0.001	0.029
A × B	<0.001	<0.001	<0.001	<0.001	<0.001	<0.001	<0.001
A × C	0.18	<0.001	<0.001	<0.001	<0.001	<0.001	0.072
B × C	<0.001	<0.001	<0.001	<0.001	0.11	<0.001	0.038
A × B × C	0.004	0.037	<0.001	<0.001	0.373	<0.001	0.528

SOD = superoxide dismutase; POD = peroxidase; CAT = catalase; PAL = phenylalanine ammonia-lyase; PPO = polyphenoloxidases; MDA = malondialdehyde.

#### POD activity

Si addition, LF infestation and LF infestation time and all interactions between these three factors significantly influenced POD activity in rice leaves (Table 1). The effects of Si amendment and LF infestation on POD activity in rice leaves were similar to their effects on SOD activity (Fig 2B). Without LF infestation, Si addition alone did not change POD activity in rice leaves at all time points. With LF infestation, POD activity showed a temporal pattern similar to SOD activity. Silicon addition increased POD activety in the infested plants (+Si+LF plants) more than in -Si+LF plants by 34.2%, 34.0%, 67.4% and 259.1% at 24, 48, 72 and 96 h post infestation, respectively (*t* ≥ 3.531, *P* ≤ 0.024).

#### CAT activity

Like POD activity, Si addition, LF infestation and LF infestation time, and all interactions between these three factors, significantly influenced CAT activity in rice leaves (Table 1). In un-infested plants, Si addition did not affect CAT activity in leaves at all time points (Fig 2C). Leaf folder infestation significantly increased CAT activity in both +Si and -Si plants compared with un-infested plants except at 96 h post infestation (*F* ≥ 12.73, df = 3, 11, *P* ≤ 0.002). Activity of CAT peaked at 48 h post infestation in +Si+LF plants and at 72 h in−Si+LF plants. In infested plants, CAT activity was significantly higher in +Si plants than in -Si plants at 48 h (by 94.0%) and 96 h (by 108.9%) post infestation (*t* ≥ 4.633, *P* ≤ 0.01).

#### PAL activity

Leaf PAL activity was also significantly influenced by Si addition, LF infestation and LF infestation time and all interactions between these three factors (Table 1). PAL activity in un-infested leaves did not differ between +Si and -Si plants (Fig 2D). In +Si+LF plants, PAL activity increased significantly by 25.5%, 104.0%, 41.1% and 31.7% at 24, 48, 72 and 96 h post infestation, respectively, over that in +Si-LF leaves (*t* ≥ 4.127, *P* ≤ 0.015). In -Si+LF plants, PAL activity was significantly higher only at 24 and 96 h post LF infestation compared with that in−Si-LF plants (*t* ≥ 4.806, *P* ≤ 0.009).

#### PPO activity

Si addition, LF infestation, LF infestation time and their interactions significantly affected PPO activity in leaves (Table 1). PPO activities showed a rhythmic change pattern, with peaks at the 24 and 72 h sampling points. In -LF plants amended with Si or not, there was no significant difference in PPO activity (Fig 2E; *t* ≤ 2.277, *P* ≥ 0.085). In +LF plants, PPO activity showed significant increases in +Si treatment compared with -Si treatment at 72 and 96 h post infestation (*t* ≥ 5.425, *P* ≤ 0.006). Particularly, PPO activity in +Si+LF plants was significantly higher than that in +Si-LF plants at all time points (*t* ≥ 4.531, *P* ≤ 0.011), while a significant difference was observed only at 72 h post infestation between -Si+LF and -Si-LF plants (*t* = 3.706, *P* = 0.021).

### MDA Concentration

Leaf MDA concentration was significantly influenced by the three treatmens and all their interactions (Table 1). When the plants were not infested, the MDA concentration was characterized by a relatively flat curve and not significantly different between +Si and -Si plants (Fig 2F; *t* ≤ 2.878, *P* ≥ 0.045). With LF infestation, MDA concentration showed significant increase only at 72 and 96 h post infestation over -LF plants (*F* ≥ 153.68, df = 3, 11, *P* < 0.001). In -Si+LF plants, MDA concentration took the shape of a upward parabola and was higher than that in +Si+LF plants at 72 and 96 h post infestation (*t* ≥ 9.547, *P* ≤ 0.001).

### Soluble Protein Content

**Fig 3:**
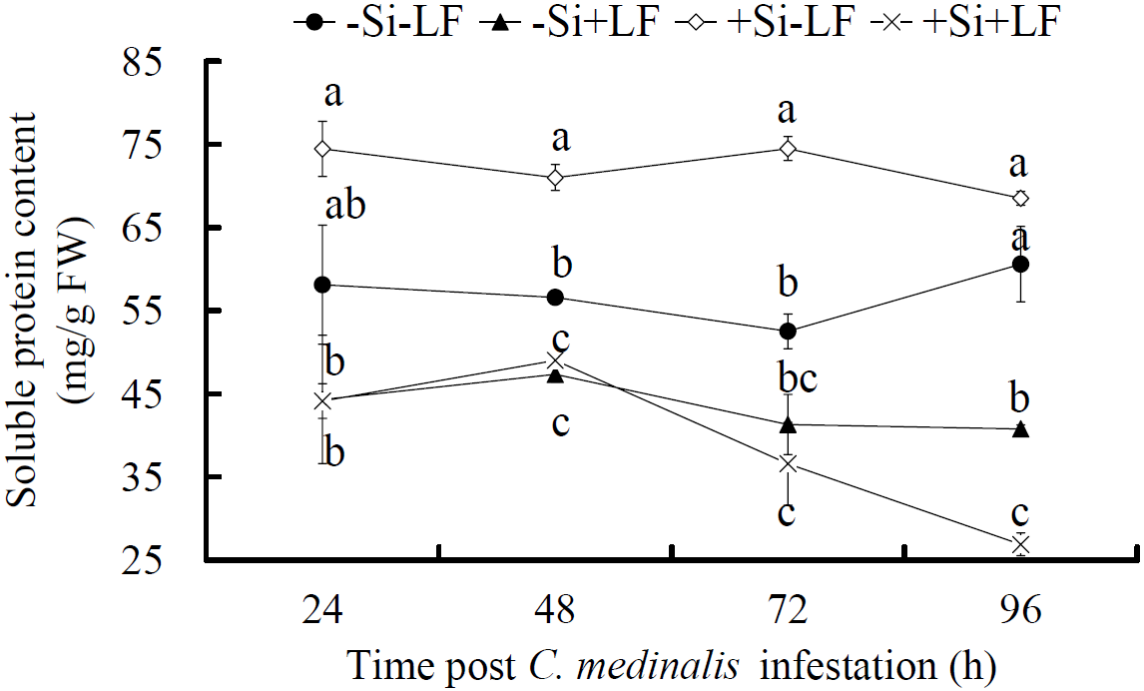
Effects of silicon amendment and *Cnaphalocrocis medinalis* infestation on soluble protein content in rice leaves. +Si = silicon amendment at 0.32 g Si/kg soil to rice plants,–Si = no silicon amendment. +LF = infestation of *C*. *medinalis* larvae,–LF = no infestation. Values are means ± SE from 3 replicates measured with different leaf samples. The means labeled by different letters at a certain time point post *C*. *medinalis* infestation are significantly different at *P* < 0.05 according to Tukey’s multiple range tests.

Soluble protein content in leaves was significantly affected by Si addition, LF infestation and LF infestation time, and also by the interactions between Si addition and LF infestation, and between LF infestation and LF infestation time (Table 1). When the plants were not infested, soluble protein content in leaves was higher in +Si than -Si plants at two of the four sampling times (Fig 3; *t* ≥ 8.427, *P* ≤ 0.001). With LF infestation, soluble protein content decreased significantly in comparison with that without infestation (*F* ≥ 6.559, df = 3, 11, *P* ≤ 0.015), and the difference between +Si-LF and +Si+LF plants (92.9 ± 23.8%, averaged over all time points) was greater in comparison with that between -Si-LF and -Si+LF plants (31.6 ± 6.1%, averaged over all time points; *F* = 8.185, df = 1,6, *P* = 0.029). Further, soluble protein content in +Si+LF plants tended to decrease at 48 h post infestation and was lower than that in -Si+LF plants at 96 h post infestation (*t* = 9.499, *P* = 0.001).

### Leaf Si Content

Si addition (*F* = 129.45, df = 1, 11, *P* < 0.001) and LF infestation (*F* = 23.42, df = 1, 11, *P* = 0.001) both significantly influenced leaf Si content at 96 h post LF infestation. Leaf Si content increased significantly by 22.6% from 7.9 mg Si/g DW in -Si plants to 9.7 mg Si/g DW in +Si plants (*t* = 10.249, *P* = 0.001), regardless of LF infestation. Leaf Si content in +Si plants increased significantly by 11.9% (*F* = 52.424, df = 3, 11, *P* < 0.001) and, in -Si plants, marginally by 5.6%, when they were infested than when they were not.

### Scanning Electron Microscopic Analysis of Silica Cells

**Fig 4:**
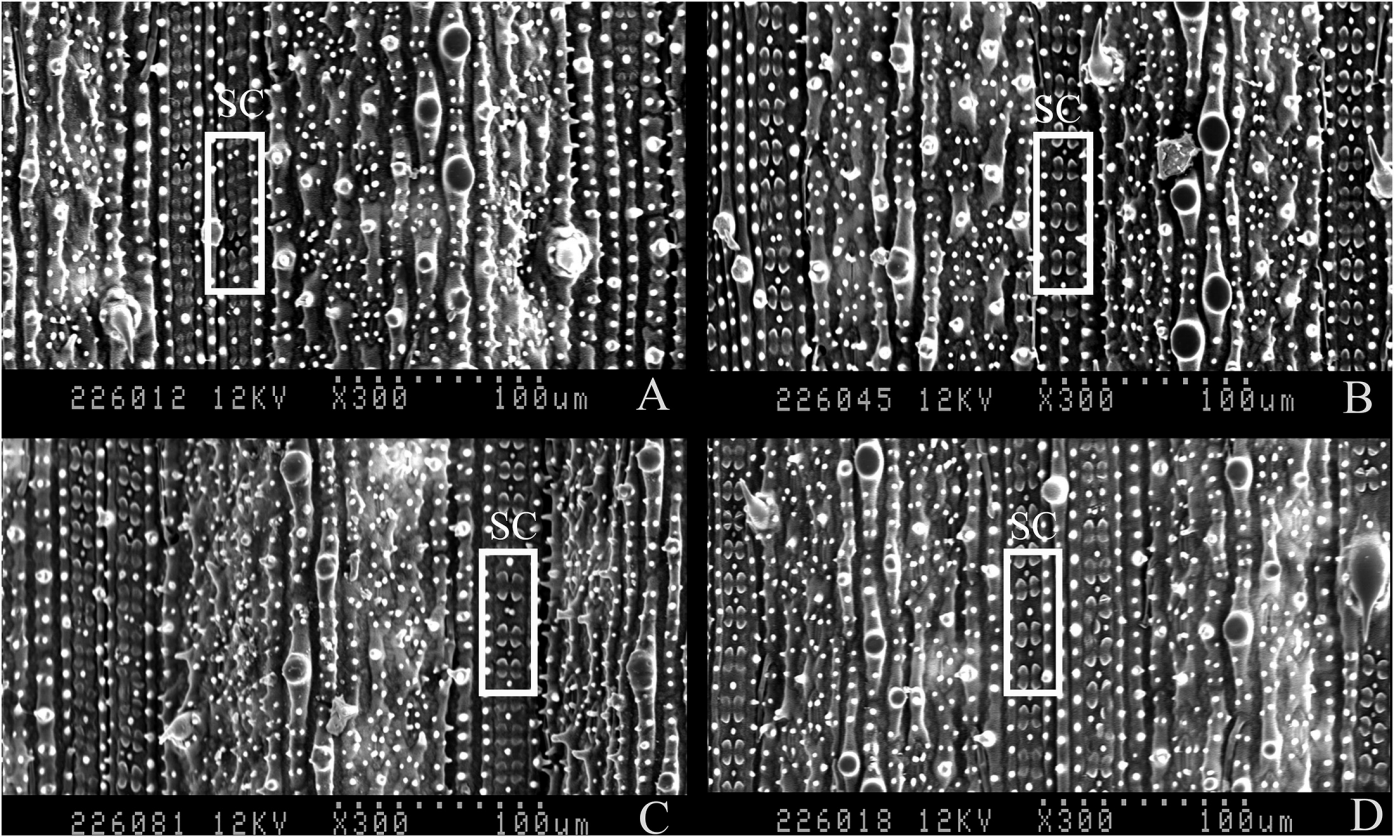
Scanning electron micrographs with 300× magnification from a cross section of rice leaf surface. The rice plants were amended with silicon at 0.32 g Si/kg soil (+Si) or not (-Si) and infested with *C*. *medinalis* larvae (+LF) or not (-LF). SC = silica cell. A: -Si-LF; B: -Si+LF; C: +Si-LF; D: +Si+LF.

The morphology of silica cells in rice leaves was observed using SEM (Fig 4). The silica cells had a dumbbell shape and were distributed in rows along the leaf veins. The results showed that silica cells varied among treatments. Regardless of LF infestation, Si addition led to intensive cell silicification in rice leaves (Table 2, Fig 4). In infested plants, rows of silica cells per 1 mm^2^ (*t* = 11.0, *P* < 0.001), number of silica cells per 1-mm row (*t* = 3.586, *P* = 0.001) and width of silica cells (*t* = 3.482, *P* = 0.001) increased significantly in +Si plants than in -Si plants by 32.4%, 4.7% and 10.0%, respectively (Table 2). For un-infested plants, the three variables were also increased significantly by 24.3% (*t* = 7.965, *P* < 0.001), 3.9% (*t* = 2.979, *P* = 0.004) and 15.2% (*t* = 4.764, *P* < 0.001) in +Si plants than in -Si plants, respectively (Table 2).

**Table 2:** Effects of silicon amendment and *Cnaphalocrocis medinalis* infestation treatments on silicification of rice leaves.

Treatments	Rows of silica cells per 1 mm^2^ leaf area	Number of silica cells per 1-mm row	Length of silica cells (μm)	Width of silica cells (μm)
-Si-LF	7.0 ± 0.15 a	55.0 ± 0.53 a	13.75 ± 0.34 a	13.08 ± 0.29 a
-Si+LF	6.8 ± 0.13 a	55.0 ± 0.59 a	13.67 ± 0.32 a	13.55 ± 0.26 a
+Si-LF	8.7 ± 0.15 b	57.1 ± 0.48 b	13.74 ± 0.26 a	15.07 ± 0.30 b
+Si+LF	9.0 ± 0.15 b	57.6 ± 0.42 b	13.61 ± 0.31 a	14.90 ± 0.29 b
n	10	50	60	60

+Si = silicon amendment at 0.32 g Si/kg soil to rice plants,–Si = no silicon amendment. +LF = infestation of *C*. *medinalis*,–LF = no infestation. Values are means ± SE. Different letters following the means in the same column denotes significant difference at *P* < 0.05 according to Tukey’s multiple range tests.

## Discussion

Rice (*Oryza sativa* L.) accumulates large amounts of silicon through an active process [37]. Silicon has been shown to play a prominent role in mediating resistance to a wide range of biotic (plant pathogens and insect pests) and abiotic stresses [5].

Interestingly, this study showed that Si addition significantly decreased the overall larval survival rate of *C*. *medinalis* through significantly reduced survival rates observed only in the first and third instars (Fig 1). The first instar *C*. *medinalis* larvae are known to be particularly susceptible to Si-mediated stress [38]. Further, the third instars have the highest consumption rate among all the instars and thus may be exposed more to the detrimental effects of Si amendment [38]. This might explain the reduced survival rates observed only in the first and third instars feeding on Si+ plants. These results confirm our previous report [11] and clearly indicate that Si-induced resistance to LF is at least partially due to cell silicification in rice leaves.

However, a physical barrier mechanism [13,14] still cannot explain the whole role of silicon in suppressing insect pests including leaf folder. Silicon appears to interact with defense-associated signaling pathways and seems to regulate a range of physiological activities in plant stress defense [15,19,39], one of which is oxidative stress resulting from overproduction of ROS by various biotic and abiotic stresses [21]. Antioxidative enzymes (SOD, POD and CAT) are important components in defense against membrane lipid peroxidation caused by ROS [40]. Silicon can enhance tolerance of plants to various stresses by altering activity of antioxidant enzymes, cation binding capacity of the cell walls, and endogenous plant hormone level [41]. In the present study, although LF infestation alone generally increased the activities of SOD, POD and CAT, the differences between +Si+LF and +Si-LF were generally great relative to those between -Si+LF and -Si-LF (Fig 2A–2C). These results indicate that the enhanced Si content in +Si plants, when triggered by LF infestation, can amplify activities of the antioxidative enzymes in rice plants, which was similar to previous reports in *Arabidopsis* plants [16], rice [32] and perennial ryegrass [42] in their responses to Si addition and disease infection. For abiotic stresses, Si alleviates salt stress in plants also by altering the production of antioxidant enzymes [41]. Therefore, it can be generalized that Si addition is involved in the priming of antioxidant enzyme systems in stressed plants.

It has been suggested that a decrease in cell membrane stability reflects high levels of lipid peroxidation caused by ROS [43]. MDA, one of the end products of lipid peroxidation, has been widely used as a biomarker of the degree of cell membrane damage [44]. In the present study, leaf MDA contents generally increased in response to LF infestation alone, but the increases were prominent in -Si+LF plants in contrast to +Si+LF plants (Fig 2F), indicating that the enhanced antioxidative enzyme activities induced by Si amendment functioned to scavenge ROS [45]. This suggests that cell membranes might be injured by LF infestation and that +Si plants might be damaged less severely compared with -Si plants.

Secondary metabolic compounds are key components in plant resistance to biotic stress [18,19]. PAL, PPO and POD are the enzymes involved in biosynthesis of secondary metabolic compounds, such as phytoalexins, phenols, and lignins [46]. Ye et al. [19] reported that POD and PPO activities did not respond to Si addition, but generally increased more in +Si+LF plants in comparison with -Si+LF plants; we found similar patterns in the activities of PAL, PPO and POD (Fig 2D and 2E). Gomes et al. [18] showed a varying pattern for the wheat-aphid system, where Si addition, infestation with aphids and their interaction all significantly enhanced PPO activity, while Si addition did not affect the PAL activity in wheat plants. In the defense responses of cucumber plants to infestation by *Pythium ultimum* [47] and *Podosphaera xanthii* [48] and of rice plants to infestation by *Magnaporthe grisea* [32], Si stimulated accumulation of polymerized phenolics and lignin by triggering the activities of PAL, PPO and POD. It can be hypothesized that the activated activities of enzymes for secondary metabolic compounds in +Si+LF plants may contribute to the reduced survival rate of *C*. *medinalis* on +Si plants, which requires further testing through measurement of contents of the secondary metabolic compounds.

Soluble protein in host plants is the main source of amino acids and an indicator of food quality for herbivores [49]. Silicon addition alone increased soluble protein content in maize plants [50]; similar results were found in this study (Fig 3). These results confirm that Si promotes plant photosynthesis [51] and stimulates protein synthesis [52]. We recorded a greater decrease in soluble protein content in +Si plants than in -Si plants due to LF infestation (Fig 3). Herbivory results in reduced soluble protein content in plants [53], which may result from reduced photosynthetic capacity due to pest damage [54] and/or vigorous synthesis of defensive enzymes and other protein-based non-enzymatic compounds in infected plants [55]. Further investigation is needed to address the activity responses of protein synthetases and proteases to Si addition and LF infestation so as to understand the mechanisms for the greater decrease in soluble protein content in +Si+LF plants than in−Si+LF plants.

In conclusion, our results show that soil amendment with Si decreased larval survival rate of *C*. *medinalis*, which may be due at least in part to the direct effects of intensified silicification of rice leaves, and particularly, to the indirect effects of activity priming of both antioxidative enzymes and enzymes for secondary metabolic compounds and decreased soluble protein content in Si amended and leaf folder infested plants. Hence, our studies have furthered our understanding of the mechanisms for the enhanced plant resistance to herbivores with Si amendment to plants, i.e. the direct defense through increased physical resistance [13,14] and indirect defense through active priming of plant chemical defenses [18,19]. Nevertheless, the metabolism of the secondary metabolic compounds and the mechanisms for the large decrease in soluble protein content in response to both Si amendment and leaf folder infestation deserve further study.

## Supporting Information

S1 Data Data obtained in the study. (XLS)
